# Macrophages as an Emerging Source of Wnt Ligands: Relevance in Mucosal Integrity

**DOI:** 10.3389/fimmu.2019.02297

**Published:** 2019-09-24

**Authors:** Jesús Cosin-Roger, Mª Dolores Ortiz-Masià, Mª Dolores Barrachina

**Affiliations:** ^1^Hospital Dr. Peset, FISABIO, Valencia, Spain; ^2^Departamento de Medicina, Facultad de Medicina, Universidad de Valencia, Valencia, Spain; ^3^Departamento de Farmacología and CIBER, Facultad de Medicina, Universidad de Valencia, Valencia, Spain

**Keywords:** macrophage, mucosal homeostasis, regeneration, fibrosis, cancer, Wnt ligands

## Abstract

The Wnt signaling pathway is a conserved pathway involved in important cellular processes such as the control of embryonic development, cellular polarity, cellular migration, and cell proliferation. In addition to playing a central role during embryogenesis, this pathway is also an essential part of adult homeostasis. Indeed, it controls the proliferation of epithelial cells in different organs such as intestine, lung, and kidney, and guarantees the maintenance of the mucosa in physiological conditions. The origin of this molecular pathway is the binding between Wnt ligands (belonging to a family of 19 different homologous secreted glycoproteins) and their specific membrane receptors, from the Frizzled receptor family. This specific interaction triggers the activation of the signaling cascade, which in turn activates or suppresses the expression of different genes in order to change the behavior of the cell. On the other hand, alterations of this pathway have been described in pathological conditions such as inflammation, fibrosis, and cancer. In recent years, macrophages—among other cell types—have emerged as a potential source of Wnt ligands. Due to their high plasticity, macrophages, which are central to the innate immune response, are capable of adopting different phenotypes depending on their microenvironment. In the past, two different phenotypes were described: a proinflammatory phenotype—M1 macrophages—and an anti-inflammatory phenotype—M2 macrophages—and a selective expression of Wnt ligands has been associated with said phenotypes. However, nowadays it is assumed that macrophages *in vivo* move through a continual spectrum of functional phenotypes. In both physiological and pathological (inflammation, fibrosis and cancer) conditions, the accumulation and polarization of macrophages conditions the future of the tissue, facilitating various scenarios, such as resolution of inflammation, activation of fibrosis, and cancer development due to the modulation of the Wnt signaling pathway, in autocrine and paracrine manner. In this work, we provide an overview of studies that have explored the role of macrophages and how they act as a source of Wnt ligands and as mediators of mucosal integrity.

## Introduction

Wnt signaling is a molecular pathway found across species and cell lines and which is absolutely necessary for development and for the growth, homeostasis, and regeneration of most tissues in an organism. This molecular cascade plays a pivotal role determining cell fate by controlling critical processes such as migration, polarity, regulation of the neural pattern, and organogenesis during embryonic development (1). Evidence of its central role in the development and homeostasis of several tissues is the fact that aberrant alterations of this molecular pathway have been involved in multiple human disorders and pathologies such as birth defects, autoimmune diseases, metabolic diseases, and cancer.

In the present review we summarize our current understanding of the main participants in Wnt signaling and the role of this pathway in mucosal homeostasis, regeneration, fibrosis, and cancer. In addition, we will describe the different sources of Wnt ligands in mucosal tissue, with a special focus on the role that macrophage-derived Wnt ligands play in physiological and pathological conditions.

## Wnt SIGNALING components

The interaction between a Wnt-secreted protein and a Frizzled receptor triggers the activation of several downstream pathways, which, in turn, has a bearing on the future of the cell and, indeed, that of the organism. One reason for the huge complexity of this pathway is the presence of multiple Wnt genes in any animal genome. In fact, in vertebrates, 19 different Wnt proteins have been identified and their expression is known to be temporally regulated during the development of the organism. Each Wnt protein is crucial, since the deletion of a given ligand from the genome triggers different phenotypes (2). Indeed, studies have demonstrated the role of specific Wnt ligands in the development of the organism. For instance, a lack of Wnt4 affects the normal development of the lung (3), whereas the absence of Wnt5a causes several alterations in the gastrointestinal tract (4).

Wnt ligands belong to a huge family of secreted glycoproteins of ~40 kDa, which are highly hydrophobic and cysteine-rich. Although it has been reported that Wnt ligands can be expressed in different cell types, one feature of these proteins is cell and tissue specificity (5). These ligands have the ability to mediate cell-cell communication not only between two cells that are in contact, but also over short distances. Wnt proteins are made in a cellular compartment called the endoplasmic reticulum. There, an acyl group from palmitoleic acid is added to Wnt by the membrane-spanning enzyme Porcupine. This modification allows Wnt ligands to become hydrophobic and, consequently, to be secreted through exocytic vesicles or exosomes into the extracellular microenvironment. Following their secretion, they can act in either an autocrine or a paracrine manner in cells, where they bind specifically to a heterodimeric receptor complex (6–8). All Wnt ligands have been classified in two groups depending on their downstream effects: canonical Wnt ligands, which induce a β-catenin dependent pathway (Wnt1, 2, 3, 8a, 8b, 10a, and 10b), and non-canonical Wnt ligands, which induce β-catenin-independent pathways (Wnt4, 5a, 5b, 6, 7a, 7b, and 11) (9).

There are molecules that inhibit the Wnt signaling pathway. These can be subdivided into: secreted frizzled-related proteins (SFRP1-5), Dickkopf family proteins (DKK1-4), Wnt inhibitory factor (Wif1), Wise/SOST, and Cereberus. The mechanism of action involved in the inhibition of this pathway varies: while SFRPs, Wif1, and Cereberus are able to sequester Wnt agonists in the extracellular environment, DKK proteins and Wise/SOST compete with Wnt agonists and prevent their binding with the receptor LRP5/6 (10).

The activation of Wnt signaling initiates when a Wnt ligand binds specifically to a cell surface heterodimeric complex composed of two components: a member of the Frizzled receptor (FZD) family and a second receptor, which can be any one of the following: the low density lipoprotein receptor-related protein 5/6 (LRP5/6), receptor-like tyrosine kinase (RYK), the Neurotrophin Receptor Homolog 1 (NRH1), the receptor tyrosine kinase like orphan receptor 2 (ROR2), or Tyrosine-protein kinase-like receptor 7 (PTK7). In vertebrates, the FZD family includes 10 different receptors (FZD1-10), all of which are composed of an extracellular region with an N-terminal sequence and a cysteine-rich domain, a seven-pass transmembrane domain and an intracellular domain with the C-terminal sequence. One of the most striking properties of these receptors is the fact that one FZD receptor can interact with different Wnt ligands to activate either canonical or non-canonical pathways. This characteristic, together with the presence of several Wnt ligands, makes it very difficult to understand this cascade (11). Indeed, the selective binding of each Wnt protein with a given FZD receptor is still poorly understood, and further research need to be performed in order to further our knowledge.

As mentioned above, Wnt signaling is classically subdivided into canonical and non-canonical pathways. In the canonical pathway, the activation of a FZD receptor and its co-receptor LRP5/6 by a canonical Wnt ligand triggers the inhibition of the complex, which destroys β-catenin due to the recruitment of the disheveled protein (Dsh) (12). This inhibition provokes the accumulation of the non-phosphorylated β-catenin in the cytosol and its translocation into the nucleus, where it interacts with Tcf/Lef transcription factors and some co-activators, thereby activating the transcription of the target genes involved in this molecular pathway (13).

On the other hand, the non-canonical pathway is initiated when a non-canonical Wnt ligand binds to a Frizzled receptor as well as with another co-receptor, which could be NRH1, Ryk, PTK7, or ROR2. In this case, the Wnt/Ca+2 or Planar Cell Polarity (PCP) pathways are activated depending on the cell type, meaning that this branch of the Wnt pathway is even more complex and less studied than the canonical pathway. Despite these differences, it is important to point out that both canonical and non-canonical pathways are crucial to essential processes such as apoptosis, proliferation, survival, cell motility, or cell fate (14).

## Wnt pathway in mucosal homeostasis

The importance of the Wnt signaling pathway in embryogenesis has been demonstrated by murine β-catenin knock-out embryos, which fail to develop the endodermal and mesodermal germ layers and are unviable (15). Wnt signaling is also required for the further differentiation of embryonic stem cells. Specifically, this pathway is temporarily regulated, so that it is activated to differentiate toward the mesoderm lineage or is inactivated to create the neuroectoderm (16).

The Wnt cascade also constitutes a key molecular pathway in the homeostasis of different tissues throughout the life cycle of adult mammals (17). In the next part of this review, we will discuss in more detail the relevance of this molecular cascade in the maintenance of mucosal integrity, specifically in the intestine and lung.

### Intestine

The intestinal epithelium is the most rapidly-renewing tissue in the body of adult mammals, regenerating every 4–5 days, while the Wnt signaling pathway is closely implicated in the homeostasis of this tissue. The first hints of the relevance of the Wnt pathway arose during genetic experiments with mice. Korinek et al. reported that the lack of TCF4, one of the central downstream effectors of Wnt signaling, caused an absence of proliferative crypts in neonatal mice (18). In line with this, the maintenance of the intestinal crypts in adult mice is dependent on Wnt signaling, since the conditional ablation of TCF4 triggers the loss of most of the proliferative crypts (19). Furthermore, the role of Wnt antagonists and Wnt agonists has also been demonstrated, with the overexpression of the Wnt antagonist DKK1 provoking a complete loss of proliferation (20). On the other hand, the transgenic expression of the strong Wnt agonist R-spondin 1 exacerbates the hyperproliferation of the intestinal crypts (21).

This rapid self-renewal is guaranteed by the presence of Intestinal Stem Cells (ISCs) at the base of the intestinal crypts. Specifically, two types of ISCs have been identified: crypt-based columnar stem cells (CBCs), which express the cell-surface marker leucine-rich repeat-containing G-protein-coupled receptor 5 (LGR5) and continually proliferate under homeostasis and an alternative reserve ISC population, which are quiescent ISCs and located in position +4 (22). Given that stem cells are located in the lower part of the crypt, Wnt signaling is present to a greater extent in the bottom of the crypt, and its activation gradually ameliorates toward the top of the crypt. It is widely assumed that Lgr5+ CBC stem cells are the driving force of tissue renewal, since they proliferate and generate a subpopulation known as transit-amplifying cells (TA cells), which quickly divide and migrate toward the upper part of the crypt, where they finally differentiate into secretory, absorptive, or enteroendocrine lineages (23–25). Therefore, as a consequence of the high rates of proliferation and differentiation among intestinal crypts, the Wnt signaling pathway coexists with several molecular pathways, such as Notch and Hedgehog, and the homeostasis of this tissue depends on the correct balance of all these pathways (26).

The intestinal stem cell niche provides the optimal environment to sustain the self-renewing and multipotent behavior of the ISCs (27). This intestinal niche is maintained due to the presence of the subepithelial mesenchyme, which is composed of myofibroblasts, fibroblasts, neuronal, and smooth muscle cells. Among all the essential factors secreted by these mesenchymal cells, Wnt ligands constitute one of the most abundant. In this way, it has been recently reported that blockage of the Wnt ligands secreted by subepithelial telocytes impairs epithelial renewal and alter intestinal integrity (28). In the same way, Wnt ligands from subepithelial Gli1+ mesenchymal cells ensure the presence of stem cells, since the specific blockage of said Wnt ligands provokes a loss of stem cells, thus altering the integrity of the intestinal epithelium and resulting in epithelial death (29).

Apart from the importance of mesenchymal cells in maintaining the intestinal stem niche, Paneth cells also secrete essential growth signals, including Wnt3, EGF, and Notch ligands. Specific Paneth are located between the CBCs, since these cells do not migrate upwards like the rest of the differentiated epithelial cells, but rather migrate downwards and constitute a niche for intestinal stem cells at the base of the intestinal crypts. The importance of these specialized intestinal epithelial cells was demonstrated by the loss of Lgr5+ stem cells due to the genetic ablation of Paneth cells *in vivo* (30). Interestingly, among all the Wnt components, these cells exhibit a pronounced and specific expression of the Wnt3 ligand. The importance of this Wnt ligand specifically from Paneth cells seems differ between *in vitro* and *in vivo* conditions. In fact, Wnt3 from Paneth cells acts as an essential niche factor for the growth and development of organoid cultures *in vitro*, while mesenchymal Wnt ligands compensate for the lack of Wnt3 from Paneth cells *in vivo* (31). In addition, Paneth cells are dependent on Wnt, which is evident in the presence of an autocrine loop. In this sense, the Wnt signaling pathway is important for the development, differentiation and maturation process of the epithelial cells that regulated the expression of the alpha-defensins HD5 and HD6 (32).

### Lung

The lung is another organ whose development and homeostasis is regulated by the Wnt pathway. In 1990 Gavin et al. identified six new members of the Wnt family that are involved in the development of the lung in the fetus (33). That report spawned several further studies using different knock-out mice of several Wnt ligands which confirmed the relevance of this molecular pathway in lung development by (34–36). Recent reports have highlighted an emerging role of the non-canonical Wnt pathway in lung development. In line with this, experiments with Wnt5a knock-out mice have demonstrated that the absence of this non-canonical ligand results in several defects in the lung, such as abnormal capillary formation and endothelial differentiation and an impaired differentiation of alveolar epithelial cells (37–39).

On the other hand, the Wnt signaling pathway is not only essential in the development of the lung, but also in lung morphogenesis and homeostasis (40). Several reports have demonstrated—by both *in vitro* and *in vivo* approaches—that the Wnt pathway is part of a tightly-honed interplay between all the different cell types present in the lung, including the epithelium, mesenchyme, and endothelium. Indeed, the lack of Wnt2, Wnt4, or Wnt7b causes lung hypoplasia (3, 34, 41). In addition, receptors such as LRP-5/6 and Wnt antagonists such as sFRP-1 and Dkk-1 have also exhibited an important role in lung morphogenesis. In this sense, the genetic deletion of LRP-5 has been shown to impair alveolar formation and angiogenesis in neonatal mice (42), whereas the accumulation of nuclear β-catenin in SFRP-1 knock-out mice increases mesenchymal proliferation and impairs alveolar formation (43).

As occurs in the intestine, the Wnt signaling pathway of the lungs manages the successful proliferation and differentiation of the stem cell progenitors located in the lung into airway smooth muscle cells (ASMCs). In fact, Cohen et al. demonstrated that Wnt7b and β-catenin control the differentiation and development of ASMCs through the Platelet-derived growth factor receptor (PDGFR) pathway (44). Although most studies demonstrate the relevance of the Wnt pathway in mice, few studies have revealed the importance of this molecular cascade in human lung morphogenesis and homeostasis. The expression of several Wnt ligands (Wnt1, Wnt2, Wnt3, Wnt4, Wnt5a, Wnt5b, Wnt6, Wnt7a, Wnt7b, Wnt8b, Wnt10b, Wnt11, and Wnt16), Wnt receptors (FZD1, FZD2, FZD3, FZD4, FZD6, FZD7, FZD8, LRP5, and LRP6), β-catenin and members of the TCF/LEF family has been detected in human epithelial cells (40).

## Wnt pathway in mucosal regeneration

One of the molecular pathways involved in the complex process of regeneration is the Wnt signaling pathway. In non-regenerating vertebrates, the Wnt pathway is active only in specific tissues with high turnover, such as the intestine, hematopoietic compartment, and epidermis, where it preserves the self-renewal of stem cells necessary for homeostasis. However, when a tissue is injured, a cascade of molecular events is activated in order to regenerate the damaged tissue as soon as possible (45). In the following section, we will review current understanding of the role of the Wnt pathway in mucosal regeneration specifically in the intestine and lung.

### Intestine

Due to its elevated turnover the intestinal epithelium has the ability to regenerate after multiple stresses or harmful agents that disrupt the tissue, such as gamma radiation, cytotoxic drugs, or after surgical resection. The process of intestinal regeneration is mainly characterized by an enhanced proliferation of the intestinal crypts. Indeed, when the intestinal tissue is damaged, the crypts close to the injury increase their proliferation in order to close the wound as soon as possible. The regenerative process has been widely studied and can be subdivided into four steps: an apoptotic phase, characterized by a continuous loss of crypts a reduction of the crypt size and shortening of the villi, a regenerative phase during which crypts close to the injury proliferate and close the wound and the normalization phase, in which the size and length of the new crypts are normalized (46).

Several studies have demonstrated that the activation of the Wnt signaling pathway plays a pivotal role in the induction of intestinal regeneration. In response to the damage, this pathway is activated and the expression of *c-Myc*, one of the most well-known Wnt targets, is increased and promotes the regeneration of the epithelial cells through Focal adhesion kinase (Fak) and Akt/mTOR signaling (47). Furthermore, the Wnt5a from the macrophages present in the mesenchyme activates the regeneration of the intestinal crypts through TGF-β signaling (48). Recently, it has been reported that Wnt2b from intestinal epithelial cells is essential for intestinal regeneration because it induces the proliferation of the subpopulation of the quiescent intestinal stem cells located at position +4 Tert+ stem cells. In the study in question, Suh et al. showed that, although the conditional ablation of these Tert+ stem cells did not alter intestinal homeostasis, tissue regeneration was significantly impaired after the injury (49). Hence, it is clear that intestinal regeneration is a complex process regulated by several non-redundant Wnt ligand sources that are crucial for the activation of the Wnt pathway and the complete regeneration of the intestine after insult.

Given the central role of the Wnt pathway in intestinal regeneration, this molecular cascade acquires a huge importance in patients with Inflammatory Bowel Disease (IBD), who suffer continuous episodes of intestinal inflammation and recovery (50). We have previously reported that this pathway is more activated specifically in the damaged mucosa of IBD patients compared with the non-damaged mucosa of the same patients. Moreover, we have shown how the activation of the Wnt signaling pathway with a Wnt agonist accelerates mucosal regeneration after acute TNBS-induced colitis (51, 52). In line with our studies, Tao et al. have recently demonstrated that microRNA-31 promotes the regeneration of intestinal epithelium following injury by regulating the Wnt signaling pathway (53).

### Lung

The epithelium of the lung can be injured by insults such as inflammation, infection, allergic reactions (asthma), physical trauma, or inhaled particles coming from cigarettes or pollen. All of these stresses provoke damage to the pulmonary epithelium, characterized by the disruption of the tight junctions between epithelial cells and impaired epithelial barrier function, which in turn leads to increased permeability, debilitated cell-cell adhesion, necrosis, and apoptosis of epithelial cells. After an injury, the regenerative mechanism is initiated in order to restore the pulmonary epithelium (54). In this context, the Wnt signaling pathway is also essential for the regeneration of the lung. It is important to consider that, although the cellular proliferation in this organ is weaker than in others, the presence of an injury still triggers the activation of this molecular cascade. The Wnt signaling pathway regulates the activity and control of lung stem cells, which differentiate toward either mesoderm or endoderm progenitor cells as a result of this molecular cascade (55). In line with this, McCauley et al. have recently published an elegantly performed study consisting of experiments with airway organoids generated from single-cell lung progenitors, demonstrating that the Wnt signaling pathway plays a central role in the regulation of the proximal and distal epithelial pattern in both murine and human pluripotent stem cells. Their results have underlined the essential role of this pathway in lung regeneration, revealing a significant reduction in tissue repair when the Wnt pathway was absent (56).

The central component of the canonical Wnt pathway, β-catenin, mediates the regeneration of the lung, thus acting as a transcription factor which activates the expression of genes involved in epithelial regeneration and regulating the tight junctions of the epithelial cells in the lung. In this respect, Cai et al. demonstrated that overexpression of β-catenin enhanced the differentiation of mesenchymal stem cells of the lung into alveolar epithelial type II cells, which improved the permeability of the lung epithelium and reduced the acute inflammation and lung fibrosis caused by administration of lipopolysaccharide (57). The activation of the Wnt pathway after injury to the pulmonary epithelium stimulates the proliferation of epithelial cells and the secretion of FGF-10, which subsequently activates β-catenin in different epithelial cells and enhances the proliferation and regeneration of the damaged epithelium (58). As previously mentioned, β-catenin also contributes to pulmonary regeneration, acting as an adherens junction protein. Finigan et al. reported that the central protein of the Wnt signaling pathway mediates pulmonary epithelial permeability, showing that the human epidermal growth factor receptor-2 (HER-2) interacts with β-catenin to trigger the dissolution of the adherens junction, reduce the cell-cell adhesion and alter pulmonary epithelial barrier function (59).

As occurs in the intestine, due to the pivotal role of the Wnt signaling pathway in the homeostasis and regeneration of the lung, this molecular cascade acquires huge protagonism in pathologies of the lung, such as pulmonary arterial hypertension (PAH), asthma and Chronic obstructive pulmonary disease (COPD) (60). GWAS studies have identified several polymorphisms in genes of the Wnt signaling pathway in asthmatic patients. Specifically, a single nucleotide polymorphism (SNP rs2929973) in the WISP1 gene has been associated with asthma in children (61). In addition, a more recent GWAS edition has identified SNPs close to the genes FZD3 and FZD6 in asthmatic patients (62). In addition to genetic analysis, an enhanced expression of some Wnt components such as WNT5a and FZD5 has been demonstrated in patients with Th2-high asthma (60).

## Wnt pathway in fibrosis

Fibrosis is characterized by an exacerbated accumulation of components of the extracellular matrix (ECM), mainly collagen fibers. This pathogenic mechanism is associated with a huge number of diseases, and can appear in several tissues, eventually culminating in organ failure. Several molecular pathways, including transforming growth factor-β (TGF-β), Notch, BMP, NF-κB, and Wnt signaling, have been associated with the development of fibrosis (63–66). In recent years, Wnt signaling has emerged as a central pathway that regulates different steps of this complex process. In addition, a profound crosstalk between TGF-β (the master regulator of fibrosis) and Wnt signaling pathways is present in both extracellular and intracellular compartments. In this regard, TGF-β and Wnt ligands can regulate each other, whereas TGF-β in the cytoplasm can induce the translocation of β-catenin into the nucleus through Smad3 (67).

In normal conditions, in response to an insult that causes damage, both epithelial and endothelial cells secrete pro-inflammatory cytokines, which leads to the formation of blood clots and a provisional extracellular matrix. Subsequently, epithelial, endothelial cells and myofibroblasts secrete matrix metalloproteinases, which degrade the basement membrane and several cytokines and chemokines that recruit and activate neutrophils, leukocytes, macrophages, T cells, B cells, and eosinophils. This initial inflammatory stage is followed by a remodeling stage during which activated myofibroblasts promote the closure of the wound. These active myofibroblasts can originate local fibroblasts recruited from bone marrow, or from epithelial or endothelial cells derived from epithelial-to-mesenchymal transition (EMT) or endothelial-to-mesenchymal transition (EndEMT), respectively (68). The persistence or chronification of these processes leads to fibrosis (69). In the next section of this review, we will describe the specific relevance of the Wnt signaling pathway in fibrosis originating in the intestine and lung.

### Intestine

Intestinal fibrosis is one of the most frequent complications in Crohn's Disease (CD) patients, and leads to an irreversible stenosis of the colon. It is believed that intestinal fibrosis in these patients is the consequence of chronic inflammation, and the therapeutic goal has been to reduce the inflammatory process. Nevertheless, anti-inflammatory drugs are incapable of resolving this complication, so surgery is currently the only option to remove fibrotic tissue, and does not necessarily prevent recurrence (70). To our knowledge, not a single study has analyzed the relevance of the Wnt signaling pathway in intestinal fibrosis. We have recently reported that, compared with non-IBD patients, Crohn's sufferers whose condition is characterized by stenotic and penetrating behavior exhibit higher levels of profibrotic markers, enhanced gene expression of FZD4 and Wnt target genes such as C-MYC, LGR5, and CYCLIN D1, and higher protein levels of β-catenin. In the same study we have also demonstrated that WNT2b activates EMT in intestinal epithelial cells through FZD4. In this way, we have demonstrated for the first time that there is an association between the Wnt signaling pathway and intestinal fibrosis, which suggests that the Wnt signaling pathway is also important in the development of intestinal fibrosis (71).

Nevertheless, the functional role of the Wnt pathway in the development of intestinal fibrosis *in vivo* is still not clearly determined. Therefore, further studies should be performed in order to analyse the specific role of Wnt signaling in each stage of intestinal fibrosis. In light of the body of evidence from studies performed in fibrosis in other organs, such as lung or kidney, we hypothesize that pharmacological inhibition of the Wnt pathway will also be beneficial in intestinal fibrosis. However, future studies are needed to confirm this hypothesis. As we will describe in the following section, there is considerable data about the role of Wnt signaling in lung fibrosis, while, as far as we know, no studies have been published about its role in intestinal fibrosis.

### Lung

Idiopatic pulmonary fibrosis (IPF) constitutes an important complication characterized by damage to the lung epithelium and activation of fibroblasts with an excessive accumulation of extracellular matrix components, all of which triggers a progressive loss of the functions of the lung. In this pathological scenario, an overactivation of the canonical Wnt/β-catenin pathway has been described in both human patients and experimental mice models. In line with this, the lungs of IPF patients exhibit an increased gene expression of WNT1, WNT7b, WNT10b, FZD2, FZD3, β-catenin, and LEF1 compared with subjects without IPF. These results have been reinforced with immunohistochemical studies which have revealed an accumulation of WNT1, WNT3a, and β-catenin specifically in the bronchial and alveolar epithelium and in myofibroblasts in IPF patients (72, 73). The functional relevance of WNT1-inducible signaling protein-1 (WISP1) was demonstrated by Königshoff et al., who reported an attenuation of lung fibrosis through WISP1 neutralization *in vivo*. In their elegantly performed study, the authors also demonstrated that WISP1 regulates alveolar epithelial cell function and the reprogramming of myofibroblasts (73). WISP1 has also been described as a downstream mediator of some profibrotic factors, such as miR-92A, Transforming growth factor β (TGFβ) and Tumor necrosis factor α (TNFα) in human lung fibroblasts, which points to a possible pharmacological target for IPF treatment (74). Of interest, the implication of the non-canonical Wnt pathway has been reported in pulmonary fibrosis. Indeed, it has been described that WNT5b activates profibrotic signaling in a β-catenin-independent pathway through the FZD8 receptor. Vuga et al. expanded these observations by demonstrating that the non-canonical Wnt ligand Wnt5a regulates the deposition of ECM by fibroblasts in the lung and protects them against apoptosis induced by oxidative stress (75). On the whole, the literature offers firm evidence that both canonical and non-canonical Wnt pathways mediate the development of fibrosis in the lung.

Given the pivotal role of the Wnt signaling pathway in the development of pulmonary fibrosis, huge efforts have been made in order to prevent the over activation of this molecular pathway by targeting this cascade at different molecular levels, with promising results having been reported to date. In this sense, the effectivenss of tankyrase inhibitors as pharmacological agents was demonstrated by Wang et al. when they reported improved survival and amelioration of the lung fibrosis induced by bleomycin after administration of the tankyrase inhibitor XAV939. In said study, the authors proposed that this inhibitor affords benefits by reducing fibroblast proliferation, impairing differentiation of myofibroblasts and enhancing differentiation of bone marrow-derived mesenchymal cells into epithelial cells (76). Cao et al. recently reported that the inhibition of Wnt signaling pathway with a peptidomimetic small-molecule inhibitor called ICG-001 impairs myofibroblast differentiation of resident lung mesenchymal stem cells and attenuates lung fibrosis (77). The therapeutic option of inhibiting the Wnt signaling pathway has recently been strongly reinforced when Chen et al. showed that blocking the Wnt pathway with a glycoprotein called *Thymocyte differentiation antigen-1* improves pulmonary fibrosis through a reduction in the proliferation of fibroblasts in cases of acute interstitial pneumonia (78).

Nevertheless, it is important to take into account that, whereas the inhibition of this pathway produces anti-fibrotic effects, the activation of the Wnt pathway in pulmonary epithelial cells during the early stages of injury is necessary in order to repair and regenerate the tissue. Therefore, further studies should be performed in order to modulate this molecular cascade, specifically at optimal moments.

## Wnt pathway in cancer

Cancer englobes a group of pathologies characterized by an uncontrolled growth of some cell types, which spread throughout the whole organism and invade different tissues. An aberrant Wnt signaling pathway has been closely related to a broad range of cancers, from their initiation to their development, due to the dysregulation of the Cancer Stem Cells (CSCs) (79). Apart from its relevance in CSCs, the Wnt pathway plays an important role in the activation of EMT in tumor cells, thus promoting cancer metastasis and progression (80).

### Intestine

Colorectal Cancer (CRC) is a frequent and lethal disease originating from an alteration of the epithelial cells that line the colon or rectum as a result of the most common mutations of the Wnt signaling pathway, which triggers an uncontrolled proliferation and migration of these cells around the organism. The first evidence of a role for Wnt signaling in cancer was published in 1991, when Nishisho et al. reported that the gene known as Adenomatous Polyposis Coli (APC), which encodes a protein implicated in the control of nuclear β-catenin, was mutated in patients with non-inherited forms of CRC (81). Since then, accumulated evidence has confirmed that an over activation of this molecular pathway leads to the formation of adenomas and, subsequently, to the development of colon carcinoma. In fact, 90% of colon carcinoma patients present acquired mutations in APC, and these mutations determine differences in the location of tumors along the large intestine (82, 83). Of interest, the reversible knockdown of APC with shRNA in a murine model of carcinogenesis promotes regression from an adenoma to normal tissue, which highlights the importance of this pathway in the maintenance of the tumor (84). A feature of CRCs that is still a mystery is the abundance of mutations specifically in APC, rather than in other WNT components. Indeed, mutations in AXIN or β-catenin genes are present only in a small fraction of CRC patients (around 5–6%) (85). This special characteristic—described only in CRC, and not in other types of cancer—needs to be addressed in the future.

The survival of these patients has increased considerably in recent years, but this has been achieved due to advances in surgery, adenoma detection, chemotherapy, and recent drugs targeting VEGF and EGFR signaling pathways. Despite the clearly pivotal role of the over activation of the Wnt signaling pathway in CRC patients and the huge amount of different strategies to block this molecular cascade, no therapy has yet obtained significant results in CRC treatment (85). The Wnt signaling pathway has been targeted at three different points: blocking the interaction between the Wnt ligand and its receptor targeting the destruction complex of the β-catenin and targeting the transcriptional activity of β-catenin. The first strategy has not obtained significant advances in CRC treatment, since most CRCs activate the Wnt pathway independently of a specific Wnt ligand. Hence, although some drugs, such as Vanticumab or Ipafricept, have progressed in clinical trials in several cancers (e.g., breast, pancreatic, and ovarian), none of them have achieved good results in CRC (86). Regarding the second strategy, some studies have shown that inhibition of Tankyrase enzymes (TNKS) improves and synergizes with approved chemotherapies and drugs which target PI3K-AKT and RAS-MAPK signaling pathways (87, 88). However, these TNKS inhibitors have a high toxicity and also interfere with the stability of proteins essential in the elongation of telomeres and cell division (89). Finally, with respect to the third strategy, the drug PRI-724, which blocks the interaction between β-catenin and the CREB-binding protein, is currently in Phase I trials, and at Phase 2 in a randomized study in which it is being used in combination with FOLFOX/Bevacizumab to treat metastatic CRC (85). As a whole, it is clear that further studies must be performed in order to identify safer molecules to target over activation of the Wnt signaling pathway, the driving force of this type of cancer. We can only hope that the huge research efforts currently being made will provide significant clinical benefits and improve the quality of life of CRC patients.

### Lung

Lung cancer is one of the most devastating forms of cancer, and can originate as one of two types depending on clinical, molecular, histological and endocrinological characteristics: small cell lung cancer (SCLC) and non-small cell lung cancer (NSCLC) (90). Unlike colon cancer, lung cancer is rarely associated with APC mutations, and the accumulation of Wnt proteins is mainly caused by dysregulation of the transcription of Wnt ligands. In this context, an exacerbated activation of either canonical or non-canonical Wnt pathway has also been detected in lung cancer, specifically in patients with non-small cell lung cancer. In fact, an accumulation of several Wnt participants, including WNT1, WNT2, WNT3, WNT5a, FZD8, Dsh, Porcupine, and TCF4, has been reported in patients with lung cancer, and this accumulation is associated with a poor prognosis (91). In addition, in certain NSCLC subtypes, a shift from the canonical to the non-canonical Wnt pathway has been detected, while both Wnt pathways have been involved in lung cancer metastasis, since the metastatic stage of lung tumors has been associated with EMT linked to β-catenin dependent signaling and the expression of the non-canonical WNT5a, which increases the expression of fibroblast growth factor (FGF) 10 and sonic hedgehog (SHH) (92, 93). Moreover, the expression of matrix metalloproteinases, whose levels increase at the metastatic stage, can be activated by both canonical and non-canonical Wnt signaling pathways (94). It is important to point out that the combination of the enhanced Wnt pathway with a constitutive activation of different molecular pathways (such as the KRAS pathway) caused by the mutations in KRAS triggers an increase in tumor size and a poor prognosis (95). The central role of the Wnt pathway in lung cancer has recently been endorsed by Wagner et al., who have just shown for the first time that activation of the canonical Wnt is involved in resistance to chemotherapy in SCLC (96).

As occurs in the intestine, given the pivotal role of the aberrant activation of the Wnt pathway in all stages of lung cancer development, several compounds have been designed and tested. These Wnt inhibitors can be subdivided into two different categories: small molecule inhibitors and biologic inhibitors. The first group of molecules includes several Wnt antagonists (e.g., ICG-001, XAV939, AV-65, PRI-724, NCTD, etc.), whereas biologic inhibitors include antibodies such as OMP-18R5, OMP-54F28, OTSA101, small interfering RNA, short hairpin RNA, and recombinant proteins such as adenoviral sLRP6E1E2 (97). The strategies used in order to block the Wnt pathway have been basically the same ones used in other cancer types, such as colon carcinoma. Several studies have demonstrated that several small molecule compounds which target β-catenin, such as XAV939, ICG-001, or TNKS inhibitors, reduce the proliferation of lung cancer stem cells (98–100). The beneficial role of some natural compounds (e.g., curcumin or 25-hydroxyprotopanaxadiol) that reduce NSCLC cell proliferation and invasion through the inhibition of the Wnt pathway has also been reported (101, 102). On the other hand, antagonist monoclonal antibodies against the Wnt ligands Wnt1 and Wnt2 have provided beneficial results in various cancers, including NSCLC (103). The positive role of monoclonal antibodies in combatting FZD receptors has also been demonstrated in lung cancer. In this context, the antibody Vantictumab (OMP-18R5), which can undermine FZD1/2/5/7/8 receptors is currently being used in combination with docetaxel in Phase I of a Clinical Trial with patients with NSCLC (104). Despite all the progress achieved in the treatment of patients with lung cancer by specifically targeting the Wnt signaling pathway, this pharmacological strategy is still at a very primary stage, and further studies must be performed in the coming years in order to improve the safety and efficacy of all these drugs. Nevertheless, the results obtained in preclinical studies and clinical trials to date endorse the Wnt signaling pathway as a promising pharmacological target in the treatment of lung cancer.

## Sources of Wnt ligands

It is logical to consider that, given the diversity of the non-pathological and pathological processes that are regulated by the Wnt signaling pathway, Wnt ligands have become the subject of study for many years. Since the first Wnt ligand was identified more than 30 years ago, several Wnt proteins have been identified, but the specific role of each ligand is still not well-characterized. Although there is a growing body of literature regarding the role of Wnt pathway components in several cell types, very little is known about the different sources of Wnt ligands and the relevance of those sources in different pathological scenarios. Therefore, we will now describe the sources of Wnt ligands and will focus specifically on macrophages as a source of Wnt ligands, mainly in inflammatory conditions.

Epithelial cells constitute an important source of Wnt ligands in several tissues, including intestine, bone, and kidney. In the intestine, the role of epithelial-secreted Wnt ligands remains controversial several reports demonstrate that these epithelial Wnt ligands are redundant and non-essential (31, 105, 106), while a recent study from Zou et al. has demonstrated that Wnt proteins in epithelial cells are necessary for the expansion of stem cells after virus-induced villus damage (22). The authors of the aforementioned study proposed that epithelial Wnt ligands are important for the repair of the intestinal epithelium, in contrast to the redundant role of epithelial Wnt proteins in intestinal homeostasis. Epithelial Wnt ligands are essential for the maintenance of the bone, since these proteins activate the Wnt signaling pathway in osteoblasts and osteoclast precursors, thus preventing bone resorption (107). Regarding the role of epithelial Wnt ligands in the kidney, it is known that Wnt9b from epithelial cells is involved in nephrogenesis, whereas epithelial Wnt11 regulates the control of ureteric bud branching (108). However, Wnt ligands from epithelial cells not only regulate physiological functions, but are also involved in pathological conditions. In this sense, specifically in the kidney, tubular epithelial cells are the predominant source of dickkopf 3 (DKK3), which acts as a profibrotic and immunosuppressive molecule that promotes renal fibrosis (109).

Mesenchymal cells have also been identified as an important source of Wnt ligands in most tissues. In the intestine, unlike that which happens with epithelial Wnt ligands, mesenchymal-derived Wnt proteins play a key role in the regulation of the proliferative status of the stem cells (105, 110, 111). Specifically, in the kidney, the Wnt4 ligand of the metanephric mesenchyme is required for tubule formation (112). Mesenchymal cells in the lung are an important source of Wnt ligands, since stromal fibroblasts secrete, Wnt ligands, including Wnt5a, which maintains alveolar stem cells and their capacity for proliferation (113). In addition, other cellular sources of Wnt ligands have been reported, such as endothelial cells and adipocytes. In the liver, Wnt ligands from endothelial cells, specifically Wnt2 and Wnt9b, help to maintain the progenitor niche (114), while frizzled-related protein 5 secreted from adipocytes controls the white adipose tissue under metabolic stress (115).

Immune cells such as macrophages, dendritic cells (DCs), T cells, and platelets can also act as cellular sources of Wnt ligands. DCs secrete Wnt ligands, whose functions include the modulation of the immune responses mediated by B and T cells. Indeed, Kim et al. demonstrated that follicular dendritic cells secrete Wnt5a, which protects isolated germinal center B cells from apoptosis through activation of the Wnt/Ca+2 pathway (116). Reinforcing this observation, Valencia et al. reported that Wnt5a from DCs exerts a regulatory effect on both dendritic cells and T cells and increases secretion of IL-2 and IFN-γ, in an autocrine and paracrine manner (116, 117).

Platelets play important roles in inflammation and act as a source of molecules that impair the activation of Wnt signaling in different cells. Among them, DKK1 derived from platelets is the most well-characterized, having been implicated in several pathological conditions. Platelets are able to inhibit Wnt signaling pathway in alveolar epithelial cells through DKK1, thus contributing to pulmonary inflammation by inducing the adhesion of both macrophages and neutrophils to alveolar epithelial cells (118). Another pathological role for DKK1 secreted from platelets has been reported in *Leishmania major* infection, since pharmacological blockage of DKK1 with the inhibitor WAY-262611 was found to weaken the cytokine production of Th2 cells and leukocyte infiltration, which protected mice from the infection (119).

T cells, one of the key protagonists of the adaptive immune response, can also act as a source of molecules that antagonize Wnt signaling. These T cell-derived Wnt antagonists modulate the immune system, triggering changes in the immune response in several conditions. Treg cells can prevent T cell-mediated colitis through Dickkopf-1 (120), while DKK3 from T cells have been implicated in the regulation of the tolerance of CD8 T cells (121).

## Macrophages as a source of WNT ligands

Macrophages are cells involved in the innate immune response, and play a variety of roles in the inflammatory process. They are essential in the release of inflammatory mediators, but also modulate wound healing and fibrosis development. These varying functions are a result of the way these cells change their phenotype depending on the microenvironment. Accumulative evidence demonstrates that macrophages constitute a significant source of Wnt proteins in adult tissues (122–127), and the synthesis of these ligands seems to depend on their phenotype (51). These observations, joined with the fact that these cells usually accumulated in chronic inflammation, point to the relevance of macrophage-derived Wnt ligands in mucosal regeneration and in complications such as fibrosis and cancer.

Macrophages display remarkable plasticity and can adopt both pro- and anti-inflammatory phenotypes, allowing them to switch between homeostatic and tissue repair functions. The differentiation programs involved in macrophage polarization have been categorized as classic M1-type activation and alternative M2-type activation. As a consequence of several stresses, such as inflammatory pathway activation or the presence of pathogenic organisms, monocytes can adopt an M1 pro-inflammatory phenotype, exerting a relevant role in host defense, and promote a T_H_1-like immune response. M1 polarization is characterized by upregulation of cell surface activation markers and molecules involved in antigen presentation, such as major histocompatibility complex class II, CD16, CD32, CD80, CD86, and IL-1 receptor, as well as the production of a range of pro-inflammatory molecules (IL-1, IL-6, IL-12, Il-23, inducible nitric oxide synthase, matrix metalloproteinase 12, and macrophage-inducible C-type lectin). During the repair phase, M2 macrophages predominate, originating from *in situ* proliferation, differentiation from infiltrating monocytes, or phenotype switch from M1 macrophages. M2 macrophages have a wide range of functions, which include the suppression of inflammation and promotion of tissue repair. The M2 phenotype has been classified into three different M2 subpopulations (M2a, M2b, and M2c) depending on the basis of their phenotype and function. Thus, macrophages are alternatively activated *in vitro* in the following manners: a) IL-4/IL-13 are designated as M2a macrophages b) immune complexes and Toll-like receptor (TLR) and/or IL-1R agonist are designated as M2b and c) IL-10, transforming growth factor-β (TGF-β) and glucocorticoids are designated as M2c. M2 markers, including CD206, CD163, arginase 1, Dectin 1, IL-10, IL-4Ra, and TGF-β_1_, are more highly expressed during late inflammation and granulation (128).

In the following sections, we describe the role of macrophages as a source of Wnt ligands in mucosal regeneration, fibrosis and cancer. As a general observation, the synthesis of these ligands seems to be associated with an M2 macrophage phenotype, while other mediators involved in mucosal proliferation and differentiation, such as Notch ligands, have recently been associated with a subpopulation of pro-inflammatory macrophages (51, 129, 130).

### Macrophage-Derived Wnt in Mucosal Regeneration

The specific expression of Wnt ligands by the macrophage phenotype was first analyzed by our group. We demonstrated that the *in vitro* polarization of human macrophages toward a M2 phenotype by means of treatment with IL4 was associated with increased levels of Wnt1 and Wnt3a with respect to non-polarized or M1 (LPS + IFNγ-treated) macrophages (51). In addition, we detected activation of the Wnt signaling pathway in intestinal epithelial cells co-cultured with M2 macrophages. *In vitro* experiments performed with human hypoxic macrophages also showed that epithelial cells impaired autophagy through Wnt1, when they are co-cultured with hypoxic macrophages (131). In contrast to this observation, Wang et al. showed that blocking Wnt secretion, by either treatment with the IWP12 porcupine inhibitor or knockdown of WLS, did not modulate autophagy or ER stress in hepatocellular carcinoma cell lines or colorectal cancer cell line (132). It seems that the specific role played by Wnt ligands in autophagy regulation deserve further investigation but of interest, we found the co-localization of Wnt1 with CD206, a marker of M2 macrophages in the inflamed intestine of patients with Ulcerative Colitis in which an impaired autophagy was detected (51, 131).

The functional relevance of macrophage-derived Wnt in intestinal regeneration has recently been demonstrated in murine models of colitis or intestinal damage. First, we demonstrated STAT6-dependent overexpression of *Wnt2b, Wnt7b*, and *Wnt10a* in IL4-treated murine macrophages *in vitro*, in parallel with defects in mucosal regeneration in STAT6 knockout mice. We showed that the administration of a Wnt agonist—as well as transfer of polarized M2a macrophages to STAT6^−/−^ mice—activated the Wnt signaling pathway in the damaged mucosa and accelerated wound healing (52). In support of these observations, the study by Saha et al., who used macrophage-restricted ablation of Porcupine (133), demonstrated that macrophage-derived extracellular vesicle-packaged Wnts are essential for the regenerative response of the intestine to radiation.

The role of Wnt signaling in lung macrophages has been studied in infectious and inflammatory processes. It has been described that Wnt3a inhibits proinflammatory cytokine secretion of murine macrophages (134), and Wnt6 has been identified as a novel factor driving macrophage polarization toward an M2-like phenotype (135). However, other studies have highlighted the role of the Wnt5a ligand as a novel marker of inflammation with intrinsic pro-inflammatory properties (122, 136). For instance, the Wnt5a ligand regulates inflammatory cytokine secretion, polarization, and apoptosis in *Mycobacterium tuberculosis*-infected macrophages (137).

The role of macrophage-derived Wnt proteins in lung regeneration is incipient and requires further study. Hung et al. demonstrated a reduced expression of Wnt4 and Wnt16 in Trefoil factor 2 (TFF2)-deficient macrophages, and reconstitution of hookworm-infected CD11c^Cre^ TFF2^flox^ mice with rWnt4 and rWnt16 restored proliferation in lung epithelia post-injury. Their work revealed a mechanism wherein lung myeloid phagocytes utilize a TFF2/Wnt axis as a mechanism that drives epithelial proliferation following lung injury (138).

Under healthy physiological conditions, the kidney macrophage compartment includes a population of long-living tissue-resident macrophages, as well as macrophages that have differentiated from circulating monocytes produced in the bone marrow. RNA sequencing shows that acute kidney injury causes transcriptional reprogramming of macrophages resident in the kidneys, and they are enriched in the Wingless-type MMTV integration site family (Wnt). Relative expression levels measured by RNAseq have shown that levels of Wnt4 are higher than other Wnts in kidney-resident macrophages (139). In the same line, Wnt7b derived from wound-healing or pro-reparative macrophages has been shown to promote tubular epithelial cell proliferation, angiogenesis, and kidney repair. The report in question showed that kidney injury results in an up-regulation of Wnt ligands (*Wnt4, 7b, 10a*, and *10b)* in macrophages and the canonical Wnt response in epithelial cells. Furthermore, macrophage ablation during repair of the injured kidney results in a reduced canonical Wnt response in kidney epithelial cells. Indeed, compromise of Wnt receptors or conditional deletion of Wnt7b in the macrophage lineage has been shown to undermine the repair response and persistent injury (127, 140, 141).

Macrophage-derived Wnts have been shown to affect blood vessel formation by regulating VEGF and angiopoietin signaling in vascular endothelial cells (142). For instance, during eye development, macrophages secrete Wnt7b to induce blood vessel regression (143–145).

There is growing evidence regarding the role of both macrophages and Wnt signaling in infarct healing. In line with this, Palevski et al.'s recent work shows that Wntless-deficient macrophages in myocardial infarction present a unique subset of M2-like macrophages with anti-inflammatory, reparative, and angiogenic properties, and these mice exhibit an increased vascularization near the infarct site compared with wild-type mice. They conclude that loss of macrophage Wnt secretion improves remodeling and function after myocardial infarction in mice (146).

### Macrophage-Derived Wnt in Fibrosis

In the context of intestinal fibrosis we have described that CD16+ CD206+ macrophages accumulate in the mucosa of patients with Crohn's Disease, and that these macrophages express high levels of Wnt2b compared with controls and patients with a stenotic pattern (147, 148). In a similar manner, Salvador's work demonstrated the accumulation of CD16+ macrophages, a subgroup of macrophages that express Wnt6, in the mucosa of STAT6 knockout mice treated with TNBS to induce intestinal fibrosis. It seems likely that a pro-fibrotic macrophage phenotype expressing CD16 accumulates in both human and murine intestinal fibrosis, although the specific Wnt ligand expressed by these cells may differ depending on the species analyzed (147).

Although macrophages, particularly the alveolar type, are important for host defenses in the lung, they can also contribute to tissue fibrosis. The role of macrophage-derived Wnt proteins in pulmonary fibrosis has been analyzed in a co-culture system in Hou's study, which showed that M2 macrophages (IL-4–stimulated RAW cells, CD68^+^ CCR7^−^CD206^+^), but not M1 macrophages (LPS-stimulated, CD68^+^ CCR7^+^CD206^−^), promote myofibroblast differentiation of lung-resident mesenchymal stem cells through the release of Wnt7a (149).

Traditionally, macrophages have been recognized as key players in kidney fibrosis. Feng et al. have described that Wnt5a promotes kidney fibrosis by stimulating Yap/Taz-mediated macrophage M2 polarization (150). It is important to note that Wnt ligands have also been reported to modulate macrophage polarization toward a pro-fibrotic phenotype. In accordance with this, treatment of macrophages with Wnt3a exacerbated IL-4- or TGFβ1-induced alternative macrophage polarization (M2) (151), and activation of Wnt/β-catenin signaling can promote macrophage proliferation and macrophage accumulation, which may play a role in kidney fibrosis (152).

### Macrophage-Derived Wnt and Cancer

Tumor associated macrophages (TAMs) constitute one of the most abundant components of the tumor microenvironment. These macrophages originate in mononuclear cells present in blood vessels, which, after their extravasation, penetrate the tumor tissue and respond to all the signaling substances released from the tumor cells (153). Once there, TAMs polarize preferably toward the M2 phenotype, thus playing an important role in the suppression of the immune system, the promotion of infiltration, increase of tumor size, angiogenesis, and metastasis. Therefore, the abundance of these TAMs is an indicator of bad prognosis in cancer patients (154).

The relevance of TAMs in tumor progression has been widely studied, but the molecular mechanisms involved are not well-elucidated. There is growing evidence to support an important crosstalk between tumor cells and macrophages due to the action of many kinds of soluble factors, including Wnt ligands (155).

Oguma et al. demonstrated that infiltrated macrophages are needed for the development of intestinal tumors and are associated with activation of the Wnt signaling pathway. *In vitro*, they showed that macrophages can activate this cascade through the secretion of TNF-α. Although they did not confirm that macrophage-derived Wnt ligands are also responsible for this effect, they speculated that other soluble factors might also be involved, since they could only demonstrate a partial role of TNF-α in Wnt activation (156). In order to better characterize the expression pattern of of tumor-associated macrophages, Ojalvo et al. analyzed the gene expression signature of TAMs by subdividing them into two subpopulations: non-invasive and invasive macrophages. Among other pathways, they identified for the first time that the Wnt signaling pathway was increased specifically in an invasive subpopulation of TAMs that promotes tumor metastasis and angiogenesis. In addition, they highlighted a specific increase in the expression of Wnt5b and Wnt7b in this subpopulation of invasive TAMs (157). To our knowledge, the aforementioned study provided the first confirmation of an increased expression of Wnt components specifically in invasive TAMs. In line with this, the tumor-associated macrophage-derived Wnt7b ligand has also been involved in cholangiocarcinoma growth *in vivo* (158).

In line with the above studies, Pukrop et al. demonstrated that the expression of Wnt5a is enhanced in TAMs in breast cancer and lymph node metastasis, and non-canonical Wnt activation by this ligand increases the invasiveness of cancer cells (124). Of interest, another Wnt ligand has recently been associated with tumor progression. In fact, Linde et al. have just shown that intraepithelial macrophages secrete higher levels of Wnt1 and activate epithelial-to-mesenchymal transition in cancer cells, thus triggering and fuelling metastasis (159). Therefore, it is important to point out that tumor-associated macrophages promote tumor progression and metastasis through both canonical and non-canonical Wnt pathways.

On the other hand, TAMs can also activate the Wnt pathway in epithelial cells through several soluble factors other than Wnt ligands. These macrophages can activate Wnt signaling in colon cancer cells through the secretion of the proinflammatory cytokine IL-1β, thereby increasing the survival of cancer cells (160). TAMs can also activate epithelial-to-mesenchymal transition (EMT) in several cancers (e.g., breast, lung and pancreas) through the secretion of prostaglandin E2 and IL-10, which increases the translocation of β-catenin into the nucleus (161–163). Chen et al. have recently reinforced these observations by demonstrating that TNF-α derived from TAMs activates EMT and cancer stemness in hepatocellular carcinoma cells through activation of the Wnt signaling pathway (164). In line with this, it has recently been reported that tumor-associated macrophages enhance the proliferation and migration of osteosarcoma cells due to the activation of Wnt signaling through the secretion of CCL18 (165).

## Conclusion and future perspectives

The present review supports a central role of Wnt signaling in mucosal homeostasis and regeneration, but it also highlights how this pathway is involved in fibrosis and cancer, which complicated the pharmacological modulation of this cascade. Nevertheless, the inhibition of Wnt signaling has become a pharmacological strategy, and several compounds have been or are being designed in order to treat several diseases. A wide spectrum of different compounds has been tested in several cancers, including porcupine inhibitors, antibodies against Wnt family proteins, Tankyrase inhibitors, Disheveled inhibitors, TCF/beta-catenin Transcription Complex Inhibitors, Wnt co-activator antagonist, and Gamma Secretase Inhibitors (166). In the context of fibrosis, some inhibitors of CBP/β-catenin interaction, such as ICG-001 and PRI-724, have been tested, and promising results have been obtained *in vitro* and *in vivo* with animal models (167). Nevertheless, these compounds are yet to be approved specifically for fibrosis treatment in clinical trials. In spite of all the evidence accumulated, we still lack conclusive results regarding these compounds, and the potential effects of pharmacological inhibition of the Wnt signaling pathway are yet to be determined.

**Figure 1:**
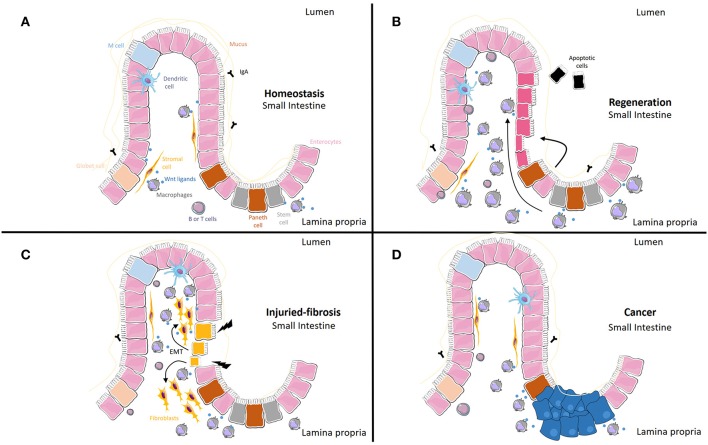
Macrophage-derived Wnt ligands and their relevance in homeostasis, regeneration, fibrosis and cancer. **(A)** Under normal conditions, macrophages act as a source of Wnt ligands. **(B)** When homeostasis is disturbed, Wnt ligands from macrophages and other cells can result in increased proliferation of epithelial cells. **(C)** Epithelial cells can transdifferentiate into fibroblasts (Epithelial-to-Mesenchymal Transition, EMT) under the influence of macrophage-derived Wnt ligands. This transition is accompanied by the progressive loss of typical epithelial cell markers and the acquisition of typical mesenchymal cell markers. **(D)** Tumor associated macrophages can activate Wnt epithelial cells through Wnt ligands and soluble factors different from Wnt ligands.

The Wnt signaling pathway is activated mainly by Wnt ligands, small proteins that are synthetized and released from different cell types. In inflammatory conditions, in which mucosal integrity is altered and macrophages accumulate, the synthesis of Wnt ligands from these cells seems to modulate mucosal regeneration, but the persistence of these ligands in chronic situations can also lead to fibrosis or cancer. In Table 1 and Figure 1, we include all macrophage-derived Wnt ligands and their relevance in homeostasis and a range of different pathologies. A better characterization of the specific ligands released from these cells, as well as the specific receptors involved in each scenario, will help to define more selective pharmacological approaches.

**Table 1:** Summary of macrophage-derived Wnt ligands and their relevance in homeostasis and different pathologies.

**Macrophage-derived Wnt ligand**	**Relevance**	**References**
Wnt1	Activates EMT and promotes metastasis in breast cancer	(159)
	M2 (IL4) macrophages act as a source of Wnt1 and promote Wnt signaling in intestinal crypts in IBD	(51)
	Hypoxic macrophages impair autophagy in epithelial cells through Wnt1	(131)
Wnt2b	M2 (IL4) macrophages overexpress Wnt2b in a STAT6-dependent manner and accelerate intestinal wound healing in mice	(52)
	M2 (CD45+CD64+CD206+CD16+) macrophages act as a source of Wnt2b and promote EMT in CD	(148)
Wnt3a	M2 (IL4) macrophages act as source of Wnt3a	(51)
	Inhibits proinflammatory cytokine secretion of murine macrophages	(134)
	Drives parenquimal regeneration of hepatocytes	(168)
Wnt4	Restores proliferative defects in post-injury lung epithelial cells produced by TFF2-deficient macrophages	(138)
Wnt5a	Enhances the invasiveness of cancer cells in breast cancer	(124)
	Regulates inflammatory cytokine secretion, polarization, and apoptosis in mycobacterium tuberculosis-infected macrophages	(137)
	Promotes kidney fibrosis by stimulating Yap/Taz-mediated macrophage M2 polarization	(150)
Wnt5b	Induces tumor progression of breast cancer	(157)
Wnt6	Up-regulated in the colonic mucosa and in CD16^+^ macrophages of STAT6 knockout mice in an IBD model	(147)
	Drives macrophage polarization toward the M2 phenotype	(135)
Wnt7a	M2 (IL4) macrophages act as a source of Wnt7a and promote lung fibrosis	(149)
Wnt7b	Induces tumor progression of breast cancer	(157)
	Promotes cholangiocarcinoma growth	(158)
	M2 (IL4) macrophages overexpress Wnt7b in a STAT6-dependent manner and accelerate intestinal wound healing in mice	(52)
	Wound-healing macrophages promote tubular epithelial cell proliferation, angiogenesis and kidney repair	(127)
	Affects blood vessel formation	(143, 144)
Wnt10b	M2 (IL4) macrophages overexpress Wnt10b in a STAT6-dependent manner and accelerate intestinal wound healing in mice	(52)
Wnt16	Restores the proliferative defect in lung epithelial cells post-injury produced by TFF2-deficient macrophages	(138)

## Author Contributions

JC-R and MO-M organized and wrote the draft manuscript. MB critically reviewed the draft. All authors contributed to the writing of the manuscript and approved the final version.

### Conflict of Interest

The authors declare that the research was conducted in the absence of any commercial or financial relationships that could be construed as a potential conflict of interest.
